# Mitochondrial dysfunction, iron accumulation, lipid peroxidation, and inflammasome activation in cellular models derived from patients with multiple sclerosis

**DOI:** 10.18632/aging.206198

**Published:** 2025-02-06

**Authors:** Raquel García-Salas, Paula Cilleros-Holgado, Anna Di Spirito, David Gómez-Fernández, Rocío Piñero-Pérez, José Manuel Romero-Domínguez, Mónica Álvarez-Córdoba, Diana Reche-López, Ana Romero-González, Alejandra López-Cabrera, José Antonio Sánchez-Alcázar

**Affiliations:** 1 Centro Andaluz de Biología del Desarrollo (CABD-CSIC-Universidad Pablo de Olavide), Sevilla 41013, Spain

**Keywords:** multiple sclerosis, iron accumulation, lipid peroxidation, inflammasome, mitochondrial dysfunction

## Abstract

Multiple sclerosis (MS) is an inflammatory demyelinating disease of the central nervous system (CNS). Despite advancements in managing relapsing active illness, effective treatments for the irreversible progressive decline in MS remain limited.

Research employing skin fibroblasts obtained from patients with neurological disorders revealed modifications in cellular stress pathways and bioenergetics. However, research using MS patient-derived cellular models is scarce.

In this study, we collected fibroblasts from two MS patients to investigate cellular pathological alterations. We observed that MS fibroblasts showed a senescent morphology associated with iron/lipofuscin accumulation and altered expression of iron metabolism proteins. In addition, we found increased lipid peroxidation and downregulation of antioxidant enzymes expression levels in MS fibroblasts. When challenged against erastin, a ferroptosis inducer, MS fibroblasts showed decreased viability, suggesting increased sensitivity to ferroptosis. Furthermore, MS fibroblasts presented alterations in the expression levels of autophagy-related proteins. Interestingly, these alterations were associated with mitochondrial dysfunction and inflammasome activation. These findings were validated in 7 additional patient-derived cell lines.

Our findings suggest that the underlying stress phenotype of MS fibroblasts may be disease-specific and recapitulate the main cellular pathological alterations found in the disease such as mitochondrial dysfunction, iron accumulation, lipid peroxidation, inflammasome activation, and pro-inflammatory cytokine production.

## INTRODUCTION

Multiple sclerosis (MS) is a debilitating neuroimmune disease affecting the central nervous system (CNS), classically characterized by demyelination and neuroaxonal degeneration [1]. Worldwide, an estimated 2.8 million people are affected by MS [2]. This disease is typically diagnosed between the ages of 20 and 50, with a higher prevalence in females than males. The main symptoms include ataxia, loss of coordination, hyperreflexia, spasticity, visual and sensory impairments, fatigue, and cognitive difficulties [3]. MS is traditionally classified into relapsing-remitting multiple sclerosis (RRMS) and two forms of progressive multiple sclerosis (PMS). RRMS patients may deteriorate into secondary-progressive multiple sclerosis (SPMS), while primary-progressive multiple sclerosis (PPMS) is characterized by neurological deterioration without early relapses [4].

The aetiology of MS remains unclear, but it is considered a multifocal demyelinating disease with progressive neurodegeneration linked to an autoimmune reaction against autoantigens [5]. Both environmental and genetic risk factors have been found to contribute to the development of MS. The autoimmune reactions in this disease likely result from the complex interplay of multiple factors over time, rather than a single causative agent. Over 200 genetic variants associated with an increased risk of developing MS have been identified, most of which regulate immune system interactions [6].

Environmental risk factors for MS include gut microbiota components, smoking, obesity, or mononucleosis resulting from Epstein-Barr virus infection, among others [7]. The primary diagnostic tool for MS is magnetic resonance imaging (MRI), which allows for *in vivo* monitoring of the CNS. A confirmed MS diagnosis typically requires the detection of two or more MRI lesions in different CNS locations, consistent with at least two clinical episodes occurring over time [5]. Additional diagnostic methods include testing for intrathecal synthesis of immunoglobulin G and analysis of cerebrospinal fluid (CSF) [6].

Currently, no blood serum biomarkers with sufficient efficiency and sensitivity exist for reliable MS detection, which could facilitate faster diagnosis [7]. Although there are no developed therapeutic agents that can fully cure MS, several medications significantly slow disease progression and alleviate symptoms [8]. MS treatment primarily involves drugs that either modulate or suppress immune function. However, despite their therapeutic benefits, these medications often have serious side effects, limiting their use [8]. Advancing our understanding of the mechanism underlying MS pathophysiology could lead to the identification of novel therapeutic targets and biomarkers, representing a significant step forward in the development of new anti-MS drugs and diagnostic methods.

To better understand the pathophysiology of MS, it is critical to identify altered pathways that impact intracellular function. In this regard, an essential component of the cell's operation is the mitochondrion. Notably, mitochondrial dysfunction has been linked to the development of various chronic illnesses, including CNS disorders [9, 10].

Given the high content of iron in mitochondria and neurons, dysregulated iron homeostasis is a known contributor to neurodegenerative diseases, including MS [11, 12]. Iron is an essential element involved in numerous physiological processes, such as oxygen transport via haemoglobin in erythrocytes [13]. In the CNS, iron contributes to neurotransmitter signalling, DNA synthesis, mitochondrial respiration, and myelin synthesis [14]. Its importance in these processes stems from its ability to catalyze redox reactions, cycling between ferrous iron (Fe^2+^) and ferric iron (Fe^3+^) ions. However, excessive iron is toxic and pro-oxidative, requiring tight regulation of its levels [11, 12]. Labile iron is highly reactive and can catalyze the formation of phospholipid peroxyl radicals, leading to cellular disruption [15] through a process known as ferroptosis [16, 17].

Ferroptosis is a form of programmed cell death characterized by iron-dependent oxidative damage, leading to lipid peroxidation and subsequent plasma membrane rupture [18]. Iron accumulation results in a progressive imbalance between antioxidant defence mechanisms and the intracellular production of Reactive Oxygen Species (ROS) [19]. Mitochondria, which contain redox transporters and enzyme complexes, are the primary sites of ROS production. These organelles have an efficient antioxidant system within their matrix to counteract the constant ROS generation [20]. Recent studies have confirmed the role of mitochondrial ROS production in promoting lipid peroxidation and triggering ferroptosis [21]. For example, in Alzheimer's disease, NADPH oxidase 4 (NOX4) induces ferroptosis in astrocytes by promoting oxidative stress-induced lipid peroxidation via the impairment of mitochondrial metabolism [22]. Emerging research also suggests a connection between ferroptosis and MS [23].

Several preclinical models replicating different aspects of the disease are used in MS research, with transgenic and humanized mouse models playing an instrumental role. The use of *in vitro* models also offers new perspectives for studying the disease [24]. Given the complexity of MS, it is essential to recognize the advantages and limitations of each animal model used in preclinical studies. Models using toxic agents, such as the cuprizone model, are valuable for studying the processes of demyelination, as they provide good reproducibility and well-defined anatomical areas of demyelination. Another widely used animal model of MS is the experimental autoimmune encephalomyelitis (EAE), which closely mimics the inflammation and neurodegeneration of the CNS characteristic of the pathology [25–27].

However, neither of the two described animal models of MS fully reproduces all the characteristic features of the disease. While the EAE effectively replicates inflammation and immune system involvement, it is not useful for studying demyelination processes. Conversely, the cuprizone model better simulates the RRMS form but has limited ability to induce chronic inflammation [28].

Skin fibroblasts from patients with neurological disorders, including MS, Alzheimer's Disease, Parkinson’s Disease, Huntington’s Disease, and Amyotrophic Lateral Sclerosis, are increasingly valuable for studying pathophysiological mechanisms and developing biomarkers [29–31].

In this study, fibroblasts derived from patients with MS were used as a cellular model to examine the main pathophysiological characteristics of the disease.

## RESULTS

### Fibroblasts derived from patients with multiple sclerosis show senescence and exhibit mitochondrial dysfunction

**Figure 1:**
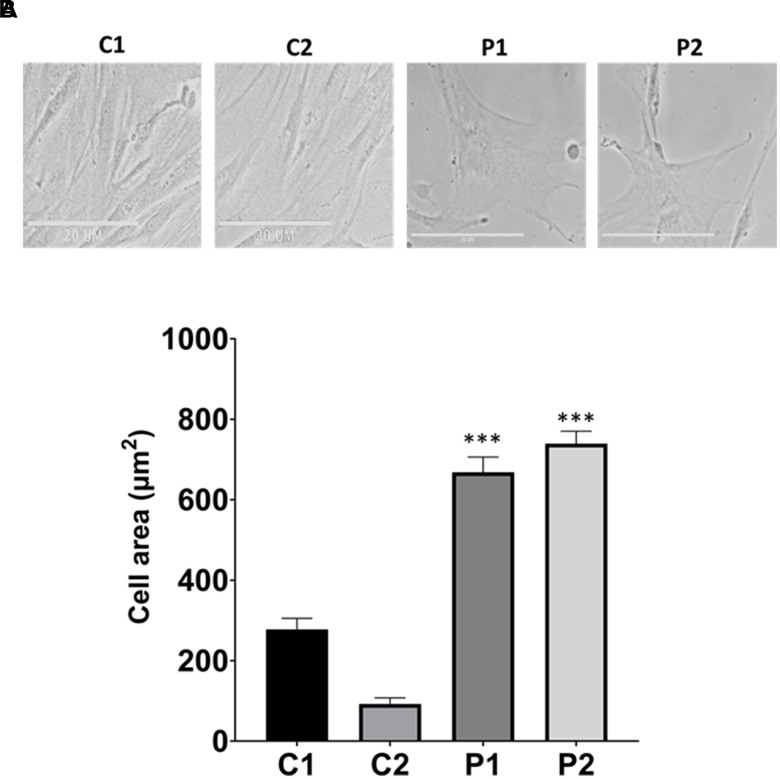
**Analysis of cell morphology in control (C1, C2) and patients (P1, P2) fibroblasts.** (**A**) Representative images of morphological characterization. Scale bar = 20 μm. (**B**) Quantification of cell area. Data represent the mean ± SD of three independent experiments. ***p-value < 0.0001 between control and MS fibroblasts.

First, we characterized the morphology of the control and patients’ cells, quantifying the cell area. Interestingly, MS fibroblasts exhibited a flattened and expanded cell shape, resembling that of senescent cells (Figure 1).

**Figure 2:**
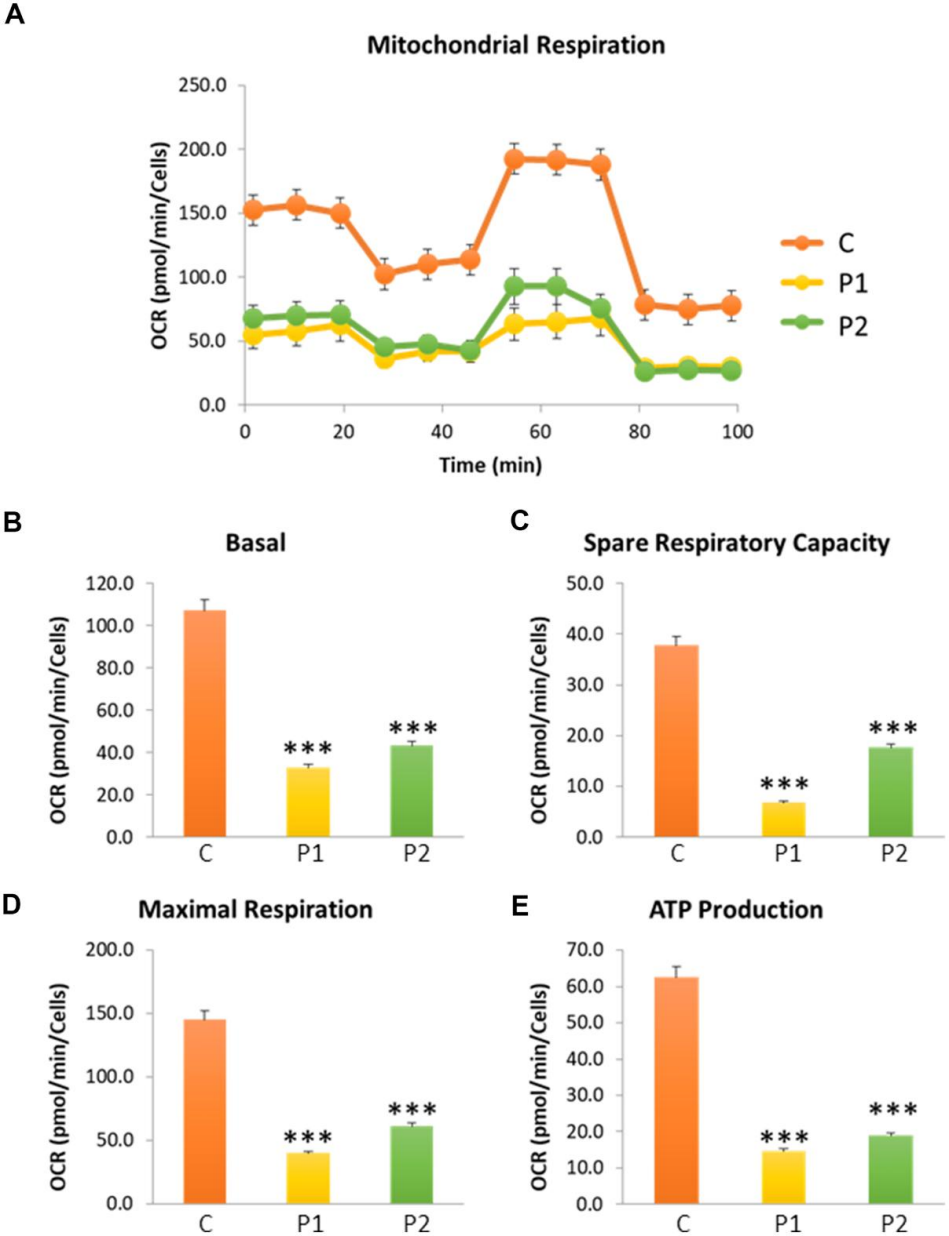
**Cell bioenergetics in control (C) and MS (P1, P2) cells. C represents the mean of C1 and C2 data.** (**A**) Mitochondrial respiration profile. (**B**) Basal respiration. (**C**) Spare respiratory capacity. (**D**) Maximal respiration. (**E**) ATP production. Data represent the mean ± SD of three independent experiments. ***p-value < 0.0001 between control and MS cells. OCR: oxygen consumption rate.

Next, we investigated mitochondrial function, which is frequently altered in neurodegenerative diseases [32]. To this end, the oxygen consumption rate (OCR) was measured in control and MS fibroblasts. Fibroblasts from patients showed reduced basal respiration, maximal respiration, and spare respiratory capacity, as well as decreased ATP production compared to control cells (Figure 2). This reduced respiratory capacity is consistent with the presence of a marked mitochondrial dysfunction.

**Figure 3:**
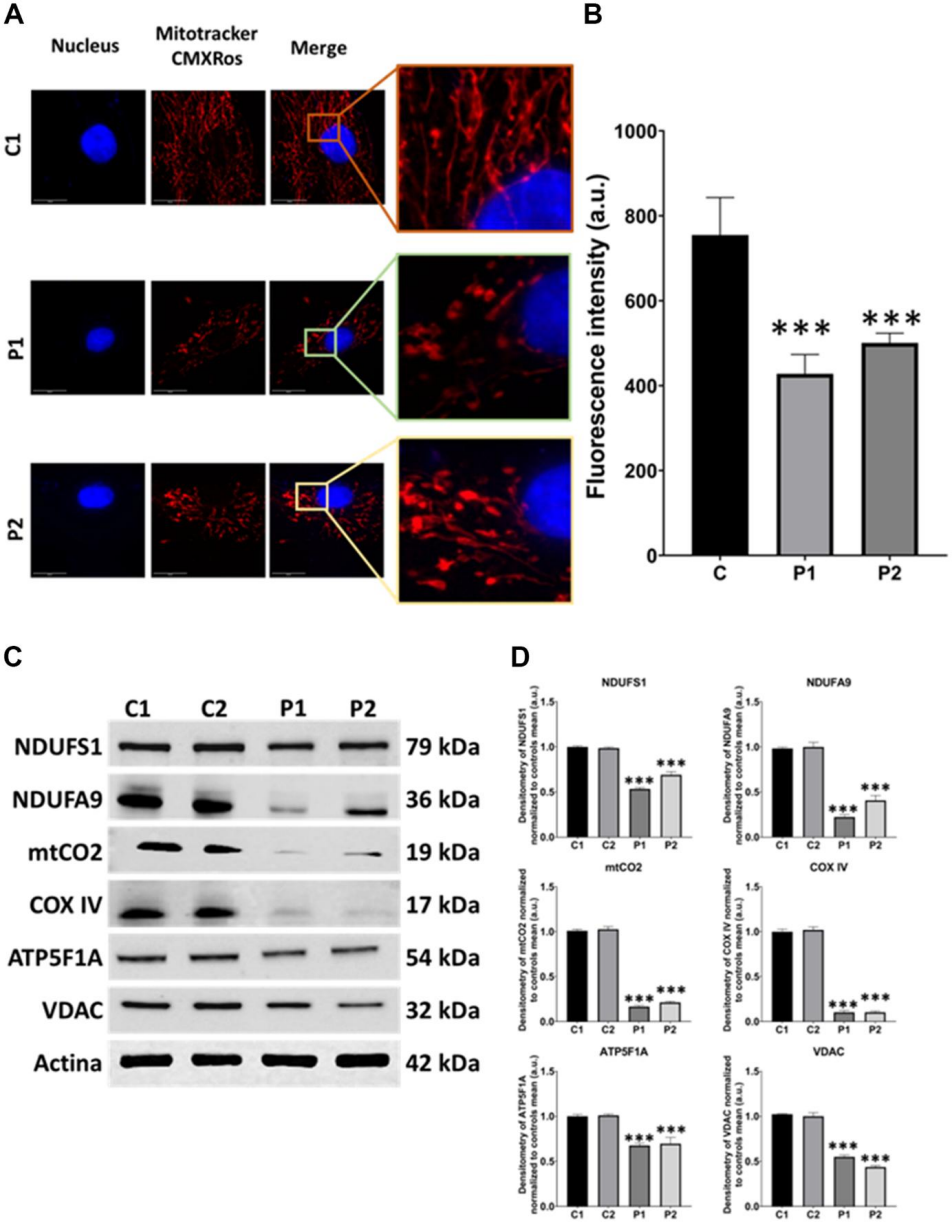
**Mitochondrial network morphology and polarization, and protein expression levels in control (C1, C2) and MS (P1, P2) fibroblasts.** (**A**) Representative images, acquired from a DeltaVision microscope. Scale bar = 20 μm. (**B**) Quantification of fluorescence intensity. C represents the mean of C1 and C2 data. (**C**) Immunoblotting analysis of mitochondrial proteins from complex I (NDUFS1, NDUFA9), complex IV (mtCO2, COX IV), and complex V (ATP5F1A). VDAC was used as a mitochondrial mass marker. Actin was used as the loading control. (**D**) Band densitometry of Western Blot data normalized to the mean of controls and referred to actin levels. Data represent the mean ± SD of three independent experiments. ***p-value < 0.0001 between control and MS cells. a.u.: arbitrary units.

We next investigated the mitochondrial network morphology by labeling mitochondria with Mitotracker^™^ Red CMXROS. The mitochondrial network of MS cells exhibited depolarized and fragmented mitochondria in comparison to control fibroblasts. We observed that the fluorescence intensity was lower in patients’ cells than in control fibroblasts, further supporting the presence of mitochondrial dysfunction (Figure 3A, 3B). Moreover, we performed immunoblotting analysis of mitochondrial proteins from different complexes, including NDUFS1 and NDUFA9 from complex I, mtCO2 and COX IV from complex IV, and ATP5F1A from complex V. VDAC was used as a mitochondrial mass marker. Our results revealed a significant reduction in the expression levels of all analyzed mitochondrial proteins in patient-derived fibroblasts compared to control fibroblasts (Figure 3C, 3D).

### Fibroblasts derived from patients with multiple sclerosis show accumulation of iron in the form of lipofuscin and alterations in the expression levels of proteins related to iron metabolism

**Figure 4:**
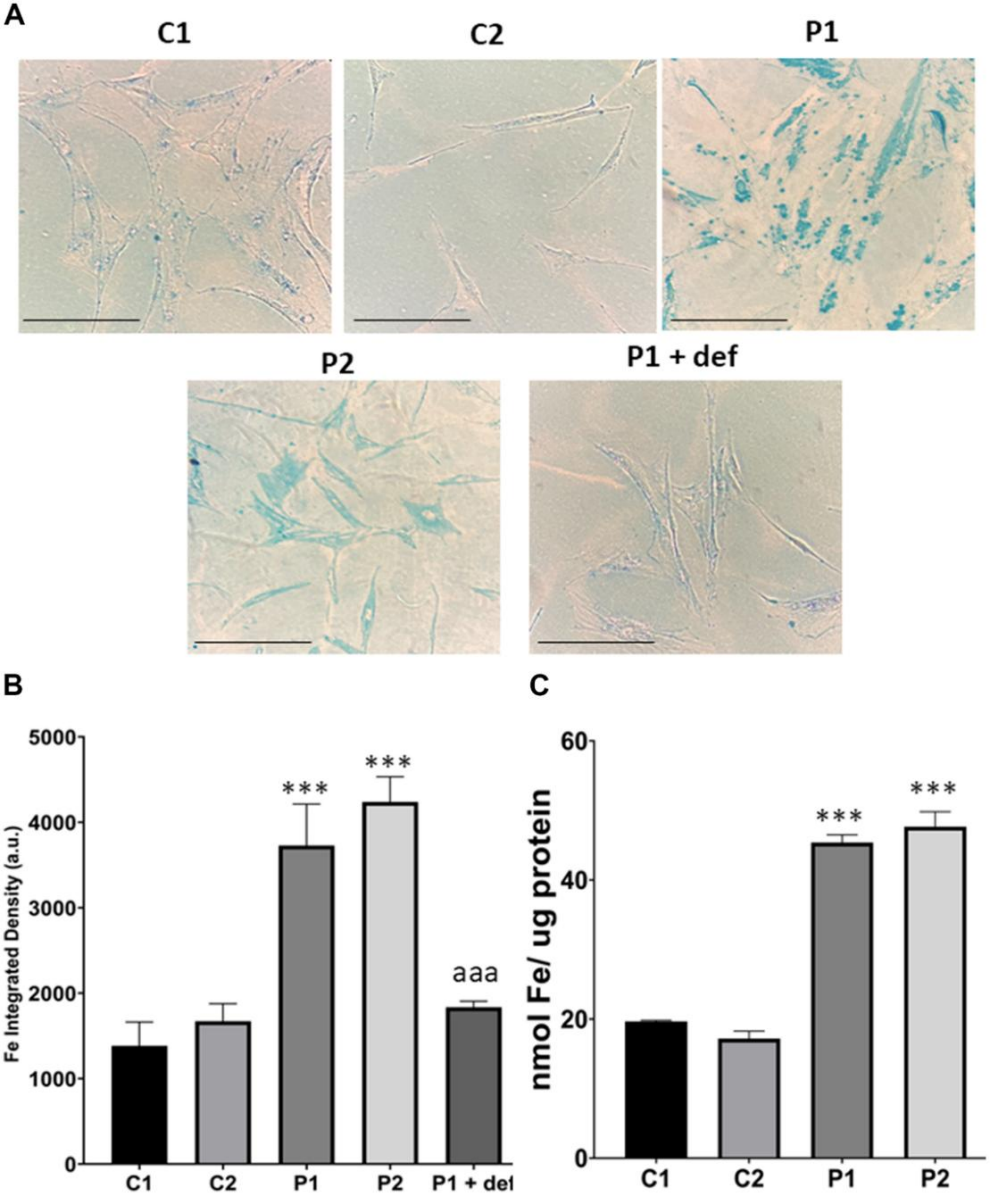
**Iron accumulation in control (C1, C2) and MS (P1, P2) fibroblasts.** (**A**) Representative images of Prussian Blue staining. Scale bar = 20 μm. (**B**) Quantification of iron integrated density. (**C**) Iron content measured by ICP-MS. Data represent the mean ± SD of three independent experiments. ***p-value < 0.0001 between control and MS cells. *^aaa^p-value < 0.0001* between untreated and deferiprone-treated P1 cells. a.u.: arbitrary units.

Next, we used Prussian Blue staining to assess iron accumulation in fibroblasts derived from MS patients. We observed that MS cells exhibited increased intracellular iron compared to control cells. Furthermore, we used P1 cells treated with deferiprone at 100 μM, an iron chelator, as a negative control (Figure 4A, 4B). To confirm the abnormal cellular iron content in MS fibroblasts, we determined the intracellular iron levels by inductively coupled plasma mass spectrometry (ICP-MS). Patients’ fibroblasts displayed a significant increase in total iron content in comparison to control cells (Figure 4C).

**Figure 5:**
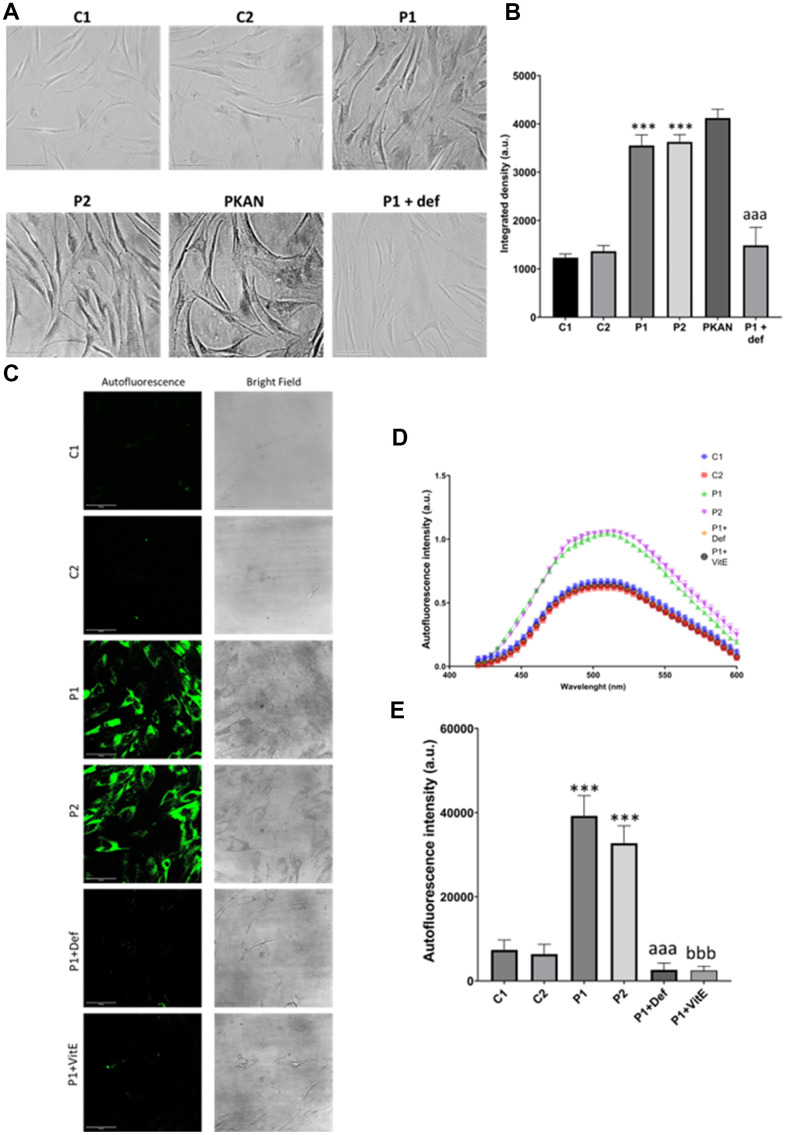
**Lipofuscin accumulation in control (C1, C2) and MS (P1, P2) fibroblasts.** P1 cells were treated with 100 μM deferiprone (P1 + def) to confirm the dependence of lipofuscin on iron. P1 cells were treated with 50 μM vitamin E (P1 + VitE) to confirm the dependence of lipofuscin accumulation on lipid peroxidation. (**A**) Representative images of Sudan Black staining. Scale bar = 20 μm. (**B**) Quantification of integrated density. (**C**) Representative autofluorescence and bright field images. Scale bar = 20 μm. (**D**) Autofluorescence spectra of lipofuscin granules measured by confocal laser scanning microscopy. (**E**) Quantification of autofluorescence intensity. Data represent the mean ± SD of three independent experiments. ***p-value < 0.0001 between control and MS cells. *^aaa^p-value < 0.0001* between untreated and deferiprone-treated P1 cells. *^bbb^p-value < 0.0001* between untreated and vitamin E-treated P1 cells. a.u.: arbitrary units.

As iron can be accumulated in the form of lipofuscin granules, we next examined the presence of lipofuscin by Sudan Black staining and autofluorescence in control and MS cells. Patient-derived cell lines showed increased autofluorescence and Sudan Black staining in comparison to the control cells, suggesting lipofuscin accumulation. Autofluorescence and Sudan Black staining in P1 cells were significantly reduced after treatment with 100 μM deferiprone, suggesting that iron was contributing to the increased autofluorescence and Sudan Black-positive lipofuscin-like material (Figure 5). Furthermore, to confirm the lipofuscin-like characteristics of the aggregates, the fluorescence spectral characteristics of lipofuscin granules in control and MS cells were analyzed by confocal laser scanning microscopy. Under excitation at 405 nm, lipofuscin granules showed an emission peak at 520-540 nm (Figure 5D).

**Figure 6:**
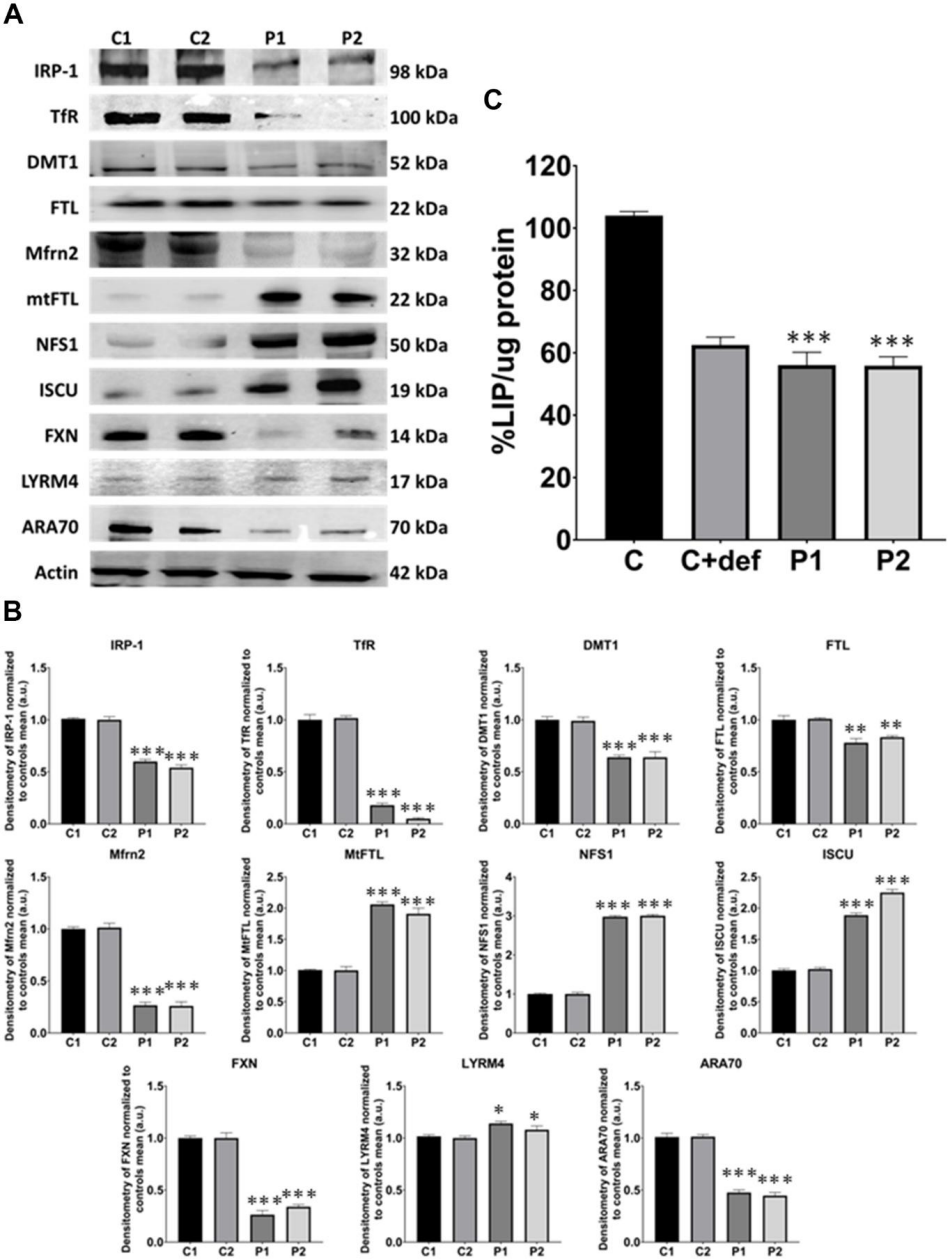
**Iron metabolism analysis in control (C1, C2) and MS (P1, P2) fibroblasts.** (**A**) Immunoblotting analysis of proteins implicated in iron metabolism. Actin was used as the loading control. (**B**) Band densitometry of Western Blot data normalized to the mean of controls and referred to actin levels. (**C**) LIP percentage. C represents the mean of C1 and C2 data. C1 and C2 cells treated with 100 μM deferiprone were used as a negative control. Data represent the mean ± SD of three separate experiments. *p-value < 0.05, ***p-value < 0.0001 between control and MS fibroblasts.

Given the perturbation in iron metabolism and distribution observed in MS cells, we next assessed iron metabolism by examining the expression levels of key proteins involved in iron trafficking, storage, and regulation, including IRP-1, TfR, DMT1, FTL, Mfrn2, mtFTL, NFS1, ISCU, FXN, LYRM4, and ARA70. We observed altered expression levels of these iron metabolism-related proteins in patients’ cells compared to control fibroblasts (Figure 6A, 6B).

Moreover, we measured the levels of LIP by a calcein assay. These levels were lower in patients’ cells than in control fibroblasts, suggesting an alteration in the cellular management of iron. Control cells treated with deferiprone at 100 μM were used as a negative control (Figure 6C).

### Fibroblasts derived from patients with MS presented lipid peroxidation, greater sensitivity to ferroptosis and alteration of autophagy

**Figure 7:**
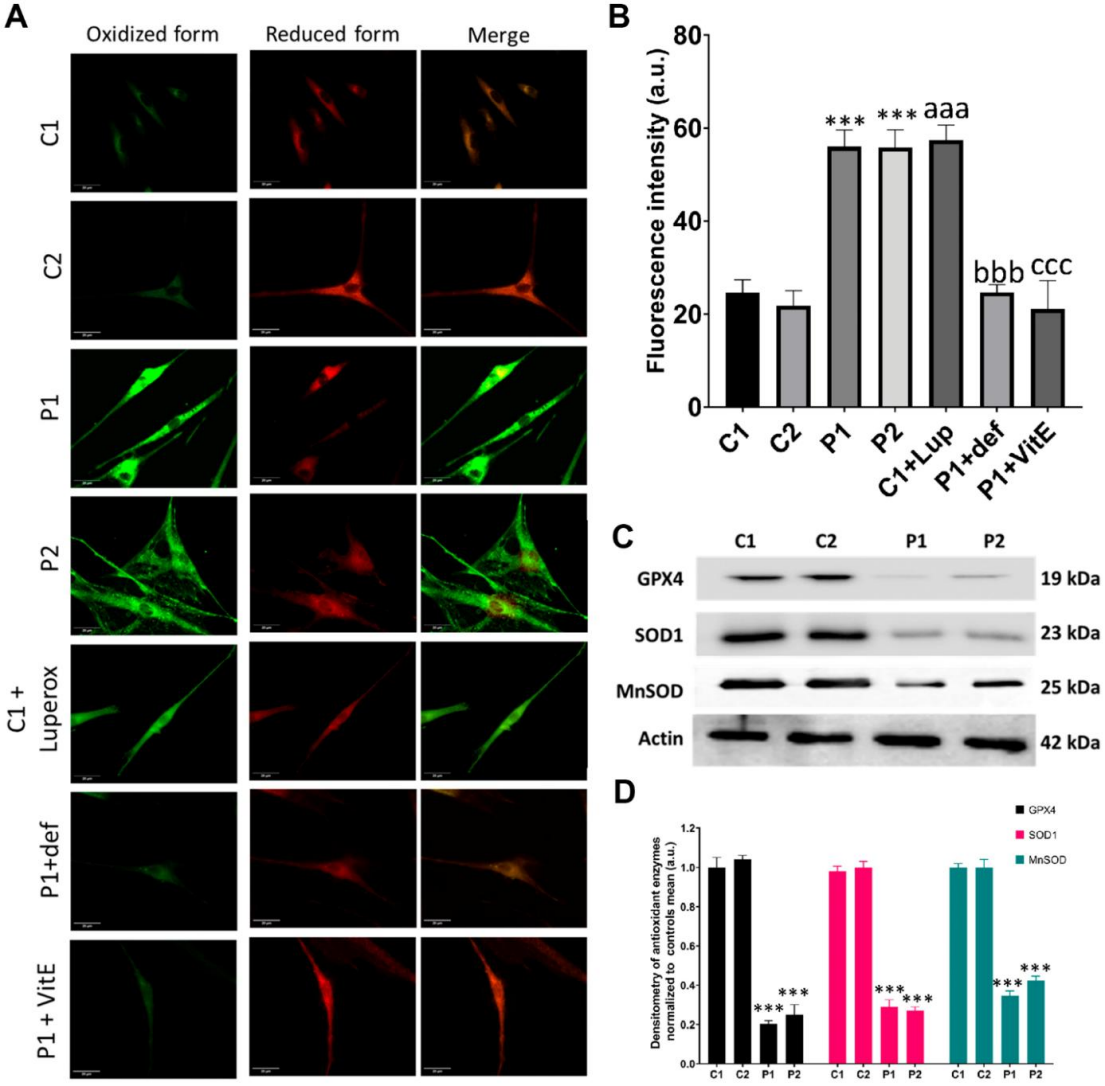
**Lipid peroxidation and antioxidant response in control (C1, C2) and MS (P1, P2) fibroblasts.** C1 cells treated with 500 μM Luperox® (C1 + Luperox) were used as a positive control. P1 cells treated with 100 μM deferiprone (P1 + def) or 50 μM vitamin E (P1 + VitE) were used as negative controls. (**A**) Representative images of lipid peroxidation assessed by Bodipy® 581/591 C11 staining. Scale bar = 20 μm. (**B**) Quantification of oxidized form fluorescence intensity. (**C**) Immunoblotting analysis of antioxidant enzymes. Actin was used as the loading control. (**D**) Band densitometry of Western Blot data normalized to the mean of controls and referred to actin levels. Data represent the mean ± SD of three separate experiments. ***p-value < 0.0001 between control and MS fibroblasts. *^aaa^p-value < 0.0001* between untreated and Luperox®-treated C1 cells. *^bbb^p-value < 0.0001* between untreated and deferiprone-treated P1 cells. *^ccc^p-value < 0.0001* between untreated and vitamin E-treated P1 cells. a.u.: arbitrary units.

Next, we examined lipid peroxidation, which often coexists with iron accumulation in neurodegenerative diseases [15]. Moreover, we assessed the expression levels of antioxidant enzymes, considering that iron overload leads to increased ROS production and oxidative stress. We observed a higher presence of peroxidized lipids in patient cells compared to control fibroblasts. C1 cells treated with 500 μM Luperox® served as a positive control, while P1 cells treated with 100 μM deferiprone or 50 μM vitamin E, a lipid peroxidation inhibitor, were used as negative controls (Figure 7A, 7B). Furthermore, we analyzed key antioxidant enzymes by immunoblotting and found a decrease in the expression levels of GPX4, SOD1, and MnSOD in patient cell lines compared to control cells (Figure 7C, 7D).

**Figure 8:**
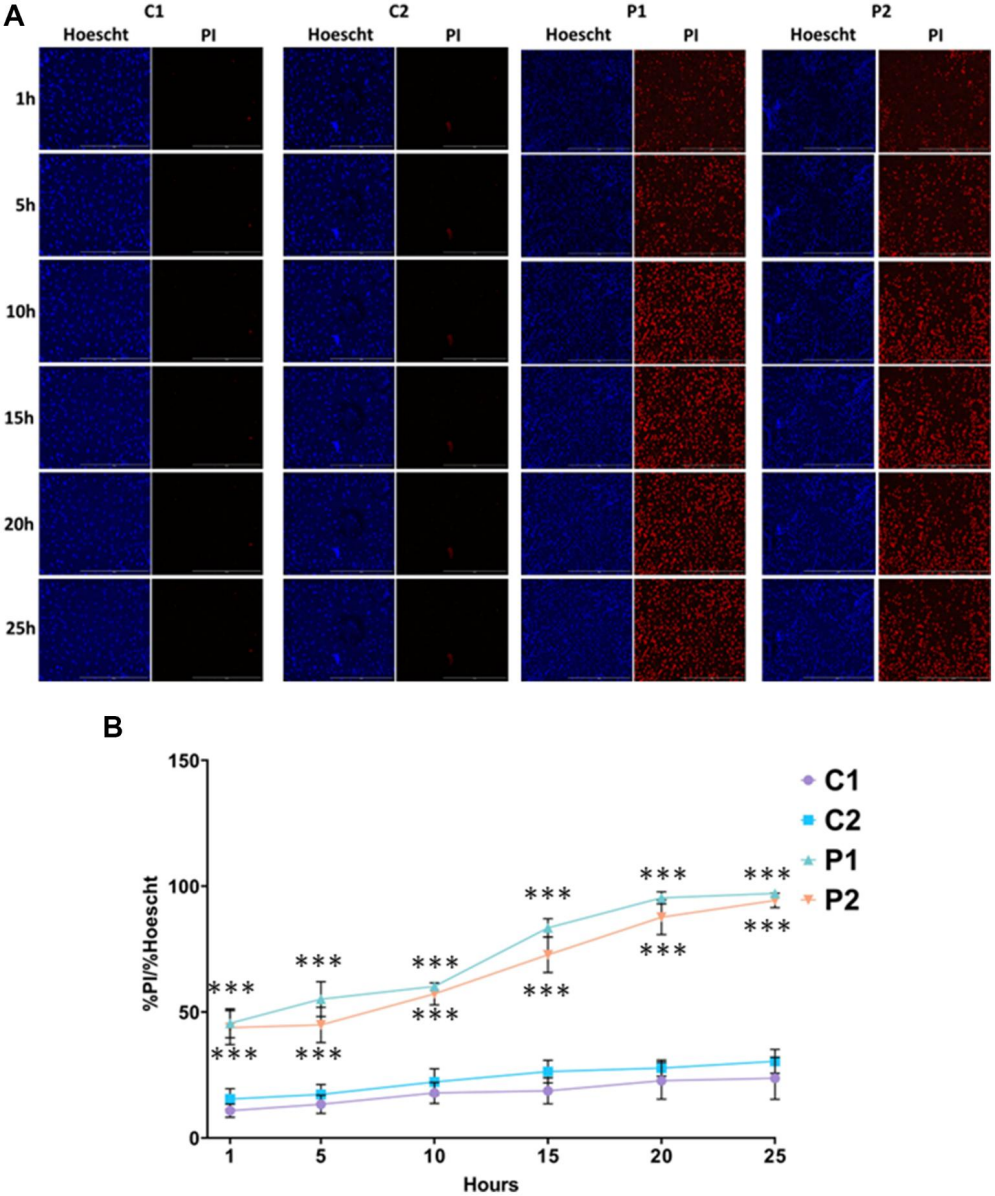
**Sensitivity to ferroptosis in control (C1, C2) and MS (P1, P2) cells.** Cells were treated with 5 μM erastin and stained with Hoechst (blue fluorescence) and propidium iodide (PI, red fluorescence) to distinguish between dead and live cells. (**A**) Representative images of live and dead cells upon addition of erastin for 25 hours. Scale bar = 20 μm. (**B**) Quantification of cell death over time. Data represent the mean ± SD of three independent experiments. ***p-value < 0.0001 between control and MS fibroblasts.

Iron accumulation and lipid peroxidation are two key factors in the genesis of a process of cell death known as ferroptosis. To evaluate ferroptosis susceptibility of MS fibroblasts, we used erastin, a known inducer of this process. Cells were treated with 5 μM erastin and stained with Hoechst and propidium iodide (PI) to distinguish between dead and live cells. We found that the number of cells undergoing ferroptosis was significantly greater in MS cell lines compared to control cells over the course of 25 hours (Figure 8).

**Figure 9:**
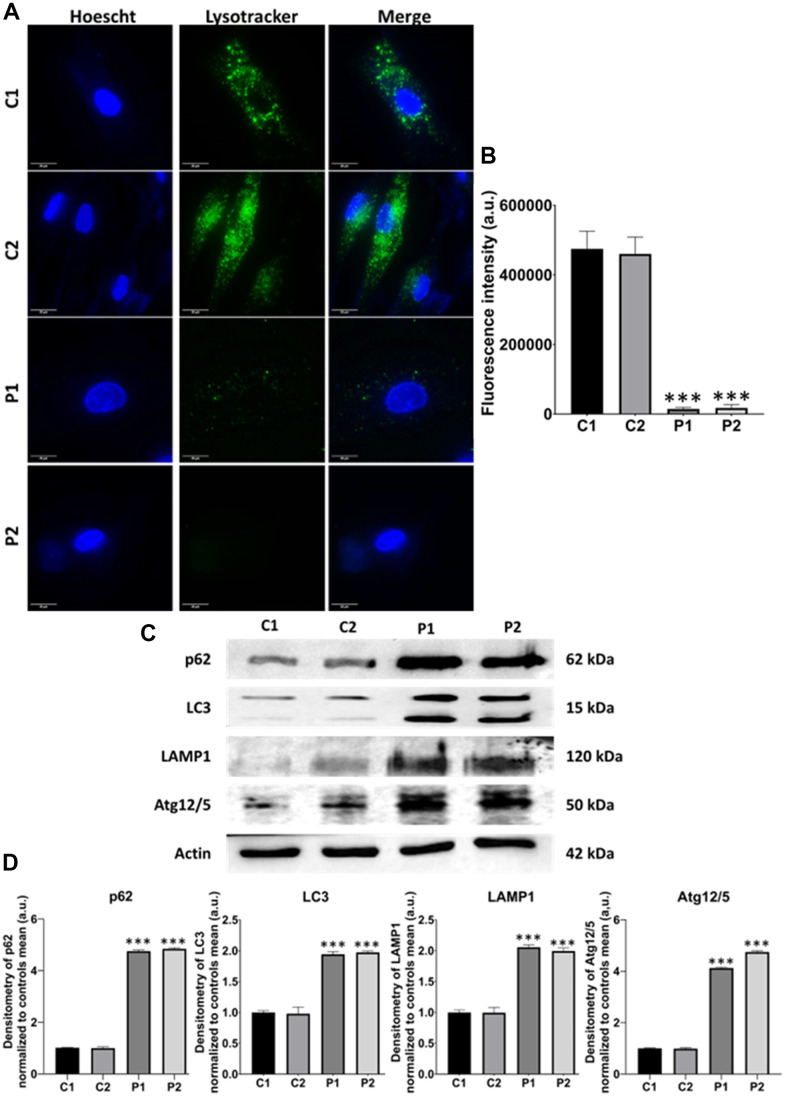
**Evaluation of autophagy in control (C1, C2) and MS (P1, P2) cells.** (**A**) Representative images of Lysotracker™ Green staining. Cells were stained with 75 nM Lysotracker™ Green for 1 hour. Nuclei were visualized by DAPI staining. Scale bar = 20 μm. (**B**) Quantification of fluorescence intensity. (**C**) Immunoblotting analysis of proteins related to autophagy. Actin was used as the loading control. (**D**) Densitometry of Western Blot data normalized to the mean of controls and referred to actin levels. Data represent the mean ± SD of three independent experiments. ***p-value < 0.0001 between control and MS cells. a.u.: arbitrary units.

We next examine another pathological alteration commonly associated with neurodegenerative diseases, autophagy [33]. For that purpose, we first evaluated the lysosomal compartment by Lysotracker™ Green staining. We observed that the fluorescence intensity was markedly lower in patients’ cells than in control fibroblasts, suggesting lysosomal acidification deficiency in MS fibroblasts (Figure 9A, 9B). Additionally, we analyzed several autophagy-related proteins by immunoblotting, including p62, LC3B, LAMP1, and Atg12/Atg5, observing increased expression levels of all these proteins in MS fibroblasts in comparison to control cells (Figure 9C, 9D).

### Fibroblasts derived from patients with MS exhibit NLRP3 inflammasome activation

**Figure 10:**
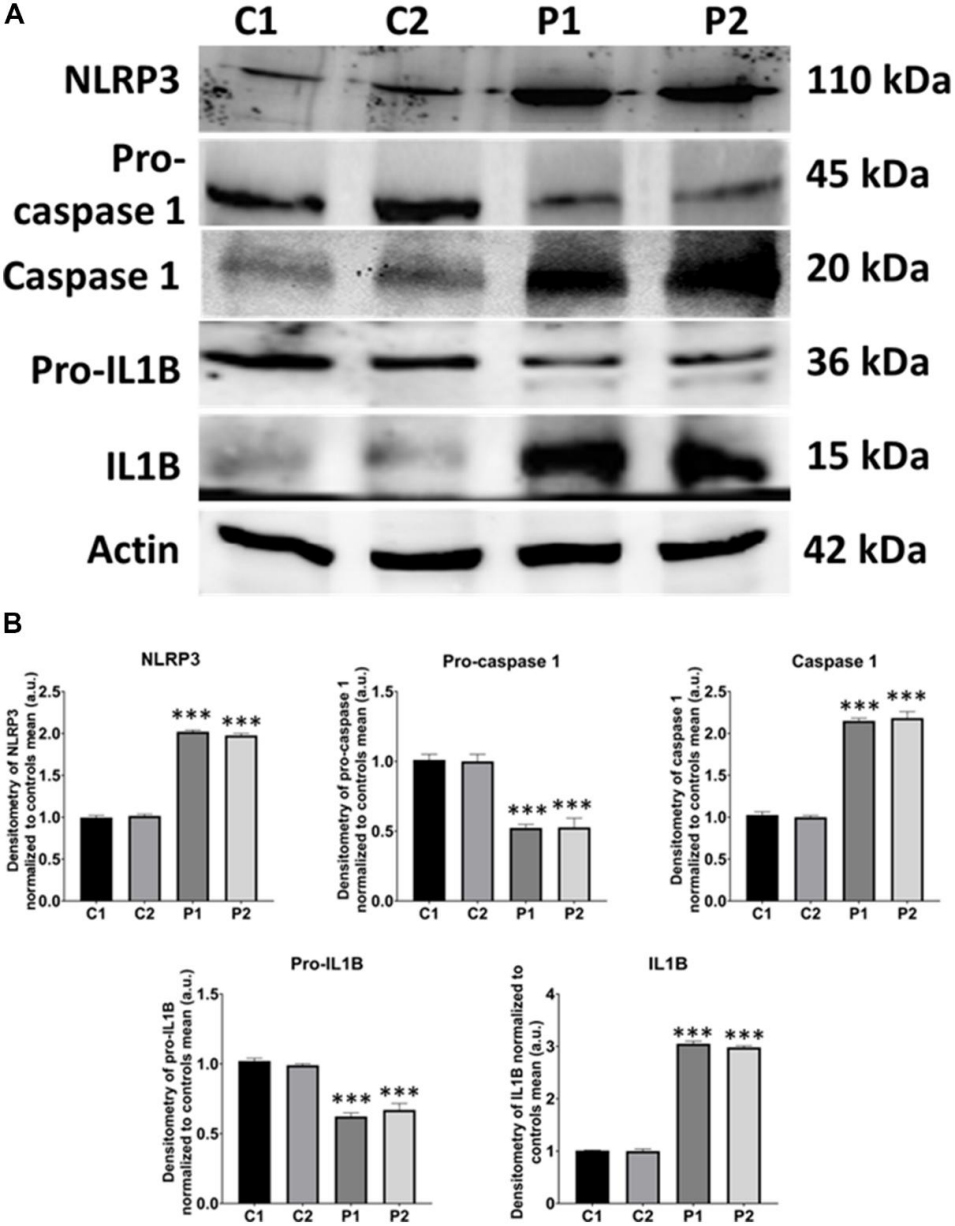
**NLRP3 inflammasome analysis in control (C1, C2) and MS (P1, P2) fibroblasts.** (**A**) Immunoblotting analysis of proteins related to NLRP3 inflammasome. Actin was used as the loading control. (**B**) Band densitometry of Western Blot data normalized to the mean of controls and referred to actin levels. Data represent the mean ± SD of three separate experiments. ***p-value < 0.0001 between control and MS cells.

Given that mitochondrial dysfunction has been implicated in activating the inflammasome, that leads to the overproduction of inflammatory cytokines and the overactivation of inflammation [34], we next examined the expression levels of NLRP3, Caspase-1, and IL1B. Interestingly, MS fibroblasts showed up-regulation of NLRP3 expression levels associated with Caspase-1 activation and increased production of IL1B (Figure 10).

### The pathophysiological characteristics were confirmed in fibroblasts derived from 7 additional MS patients

We extended our study to 7 additional patients to confirm the pathophysiological characteristics studied in the fibroblasts derived from the two patients examined previously, using cells from 4 additionally healthy control individuals.

**Figure 11:**
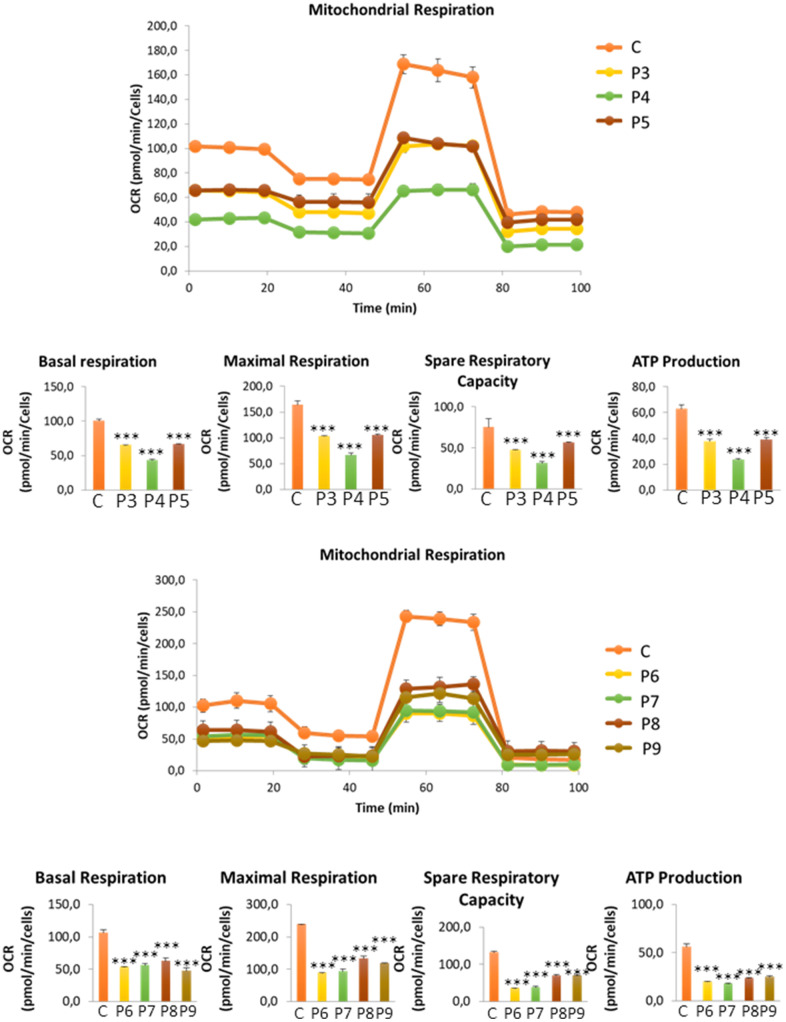
**Bioenergetics analysis of control (C3, C4, C5, C6) and MS (P3, P4, P5, P6, P7, P8, P9) cells.** Data represent the mean ± SD of three independent experiments. ***p-value < 0.0001 between control and MS fibroblasts.

**Figure 12:**
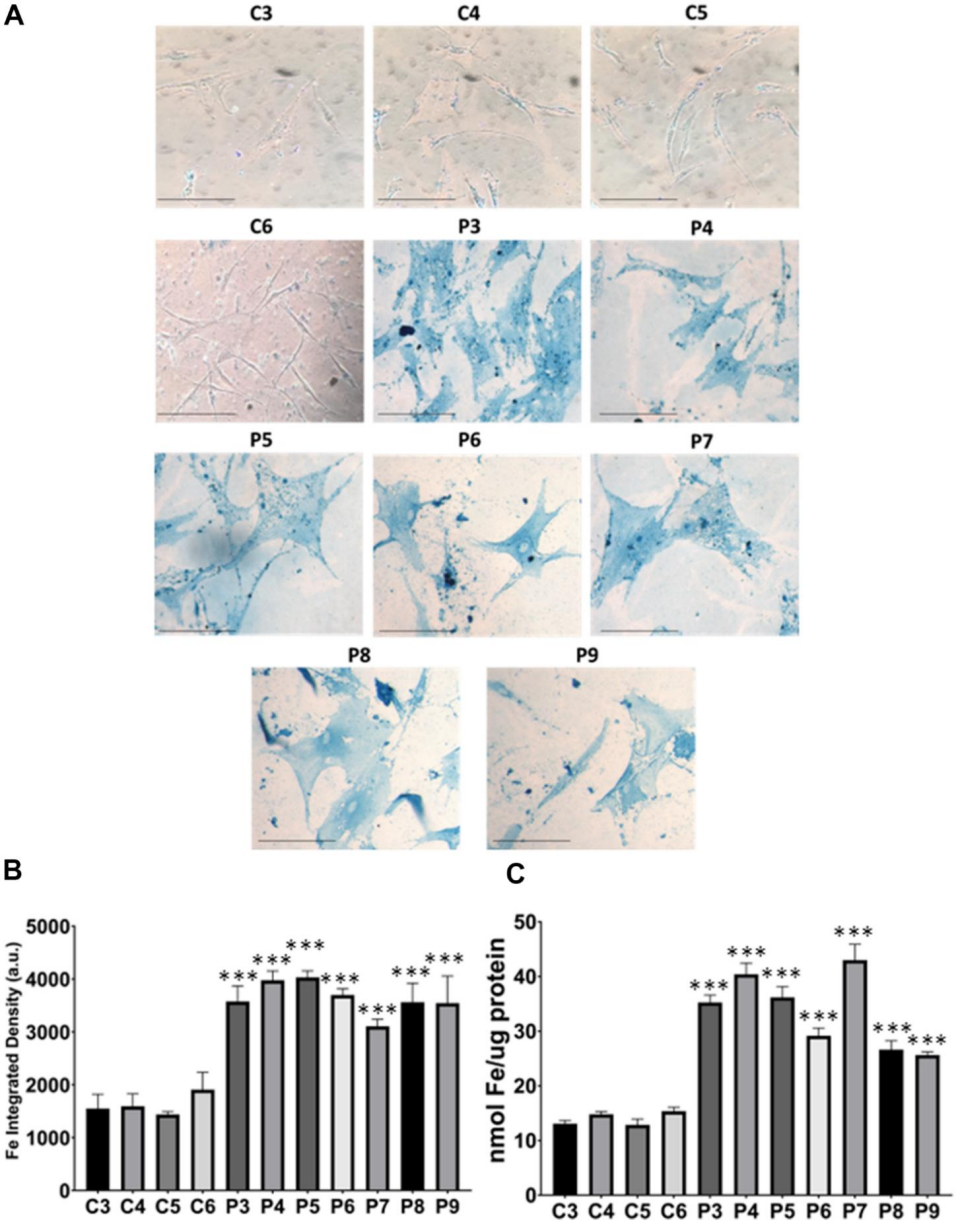
**Iron accumulation in MS cells.** (**A**) Representative images of Prussian Blue Staining in four control cell lines (C3, C4, C5, C6) and seven patient cell lines (P3, P4, P5, P6, P7, P8, P9). (**B**) Quantification of iron integrated density. (**C**) Iron levels determined by ICP-MS. Data represent the mean ± SD of three independent experiments. ***p-value < 0.0001 between control and patients’ cells. Scale bar = 20 μm. a.u.: arbitrary units.

**Figure 13:**
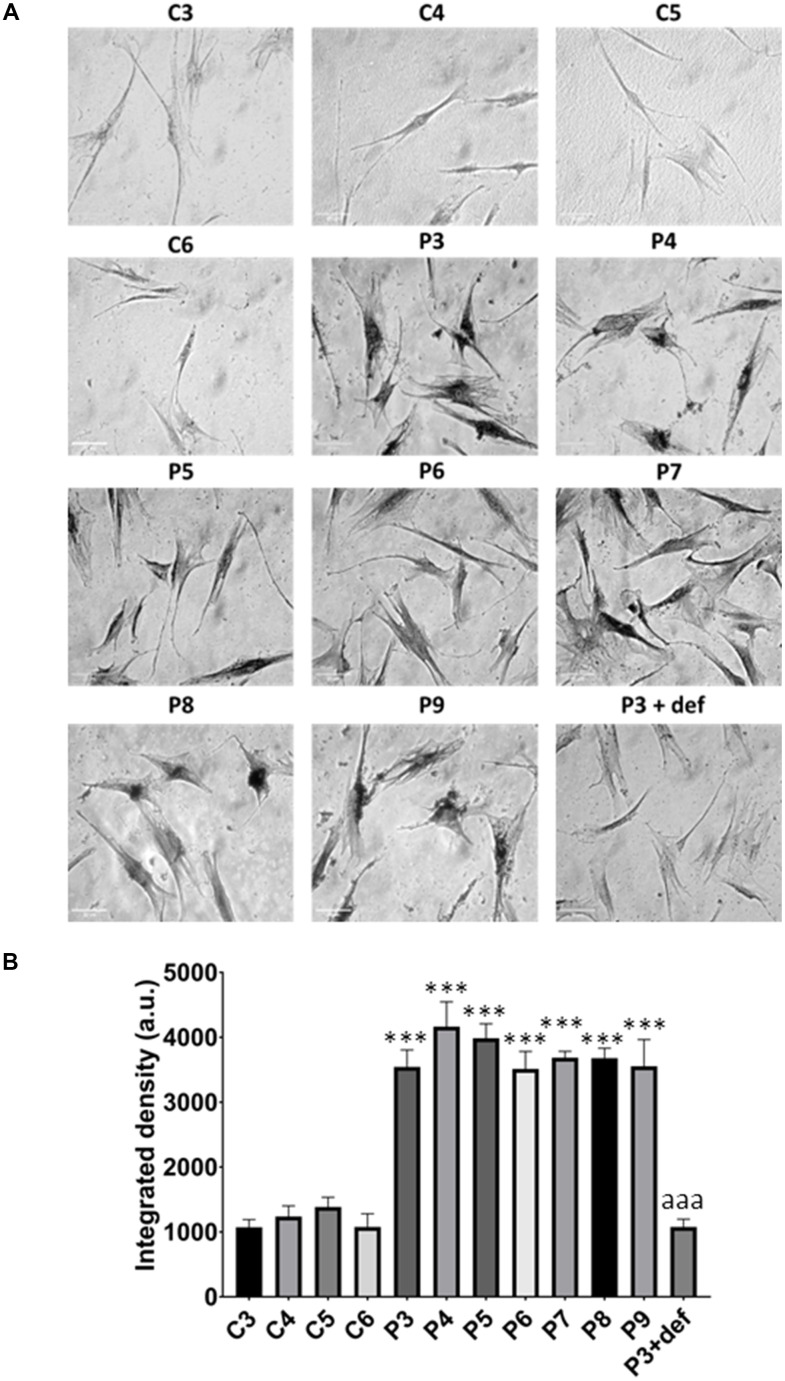
**Lipofuscin accumulation.** (**A**) Representative images of Sudan Black staining of control (C3,C4,C5,C6) and patients’ cells (P3, P4, P5, P6, P7, P8, P9). P3 treated with deferiprone at 100 μM (P3 + def) was used as a negative control. (**B**) Quantification of Sudan Black staining. Scale bar = 20 μm. Data represent the mean ± SD of three separate experiments. ***p-value < 0.0001 between control and MS fibroblasts. *^aaa^p-value < 0.0001* between untreated and deferiprone-treated P3 cells. a.u.: arbitrary units.

**Figure 14:**
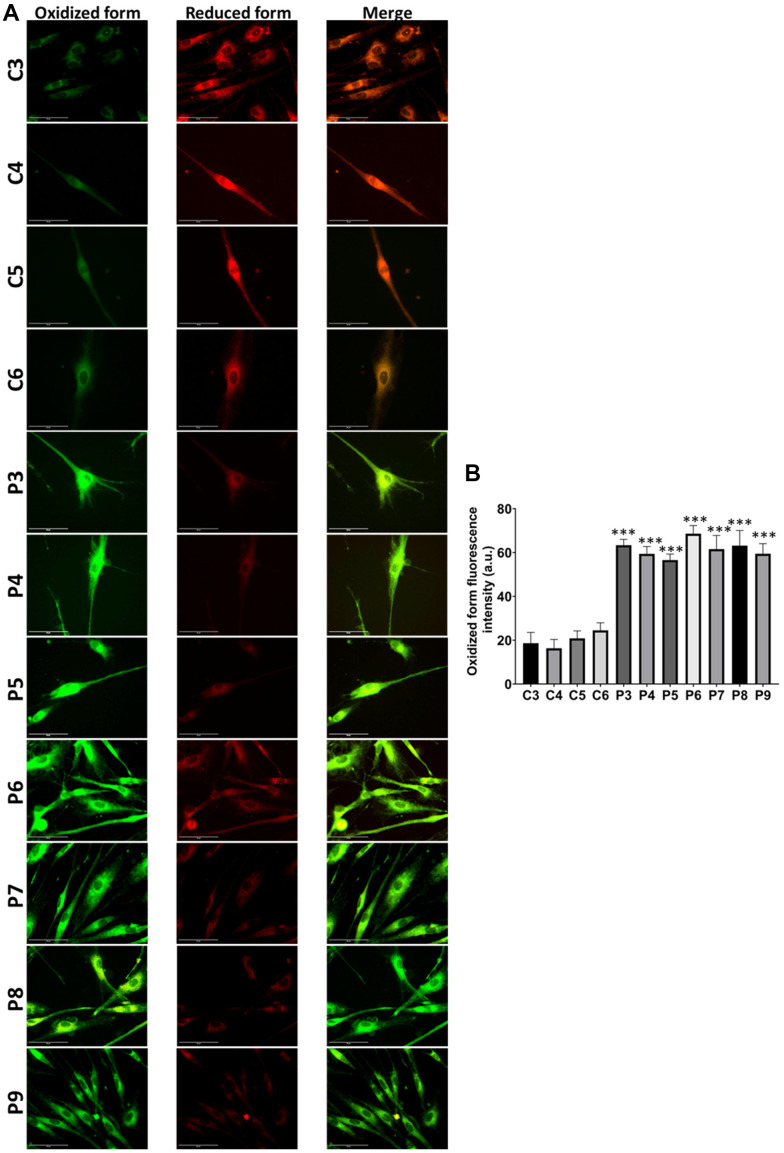
**Lipid peroxidation in control (C3, C4, C5, C6) and MS (P3, P4, P5, P6, P7, P8, P9) cells.** (**A**) Representative images of lipid peroxidation by BODIPY® 581/591 C11 staining. Scale bar = 20 μm. (**B**) Quantification of oxidized form fluorescence intensity. Data represent the mean ± SD of three independent experiments. ***p-value < 0.0001 between control and MS fibroblasts.

**Figure 15:**
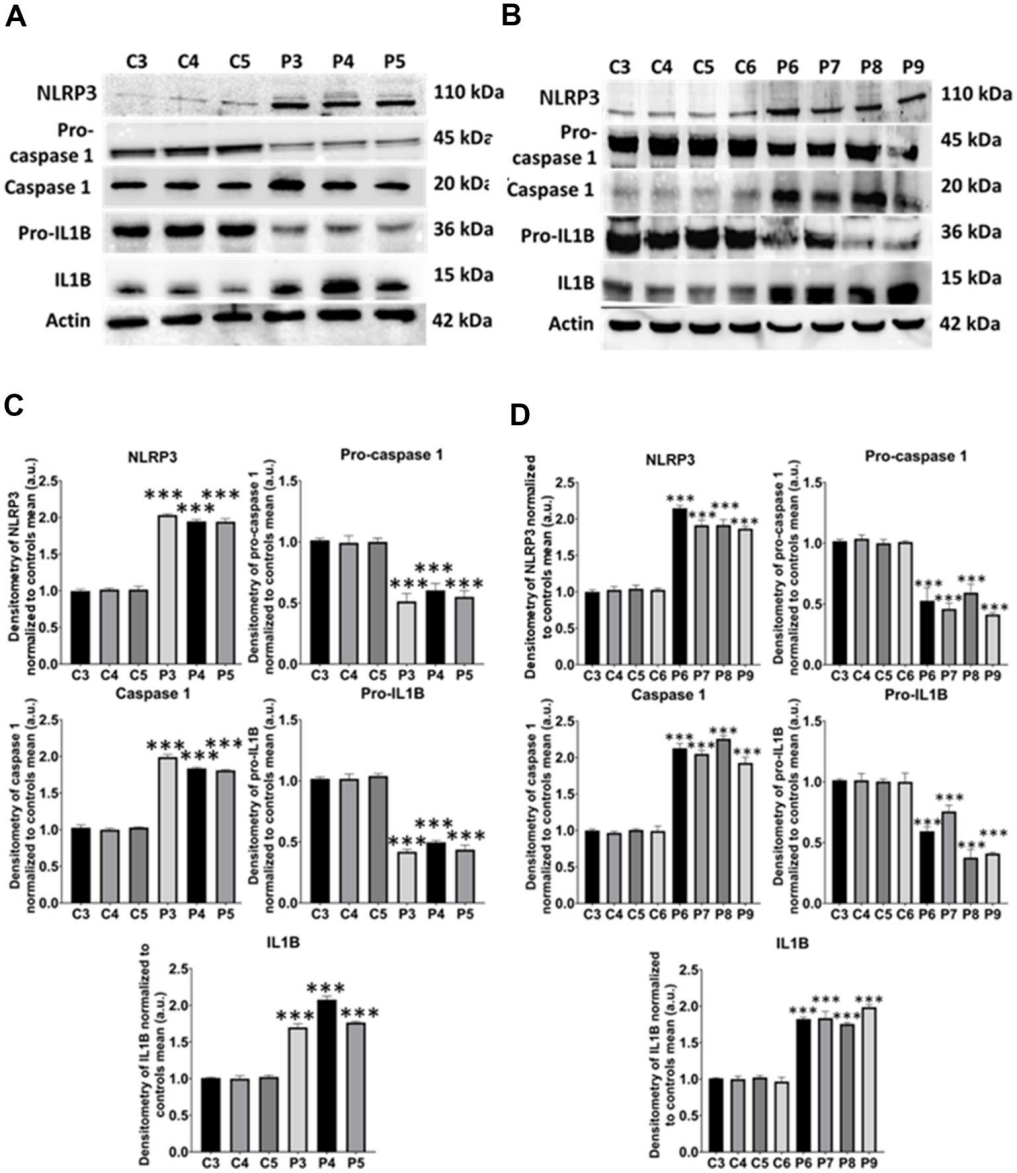
**NLRP3 inflammasome analysis.** (**A**) Immunoblotting analysis of proteins related to NLRP3 inflammasome in control (C3, C4, C5) and MS (P3, P4, P5) cells. Actin was used as the loading control. (**B**) Band densitometry of the Western Blot data normalized to the mean of controls and referred to actin levels. (**C**) Immunoblotting analysis of proteins related to NLRP3 inflammasome in control (C3, C4, C5, C6) and MS (P6, P7, P8, P9) cells. Actin was used as the loading control. (**D**) Band densitometry of the Western Blot data normalized to the mean of controls and referred to actin levels. Data represent the mean ± SD of three independent experiments. ***p-value < 0.0001 between control and MS fibroblasts.

In these additional seven patient-derived cell lines, we observed a reduction in mitochondrial respiration profile, consistent with mitochondrial dysfunction (Figure 11), as well as intracellular iron accumulation (Figure 12), lipofuscin-like material accumulation (Figure 13), lipid peroxidation (Figure 14), and NLRP3 inflammasome activation (Figure 15).

## DISCUSSION

In this study, fibroblasts derived from patients with multiple sclerosis showed a senescence phenotype and mitochondrial dysfunction, as indicated by decreased expression of mitochondrial proteins, reduced mitochondrial respiratory capacity, and mitochondrial fragmentation and depolarization. In addition, we observed accumulation of iron in the form of lipofuscin and alterations in the expression levels of proteins related to iron metabolism. These cellular alterations were further associated with NLRP3 inflammasome activation. Collectively, these findings suggest that MS patient-derived cells exhibit mitochondrial dysfunction, oxidative stress, and a pro-inflammatory phenotype.

### Mitochondrial dysfunction and MS

Mitochondrial dysfunction is increasingly recognized as a contributing factor in the pathogenesis of various chronic diseases, including MS. Mitochondria play essential roles in cellular functions such as synthesis of ATP, metabolism of several essential biomolecules, production of ROS, which serve as crucial signaling molecules, and the initiation of apoptosis and antiviral immune responses [35]. Disruptions in these mitochondrial functions can contribute to disease development.

Research on samples from MS patients and EAE mouse models has revealed several mitochondrial abnormalities. These include increased mitochondrial DNA mutations, decreased expression of mitochondrial genes, reduced activity of mitochondrial enzymes, diminished capacity for mitochondrial DNA repair, disruptions in the equilibrium of mitochondrial dynamics, and modifications in cellular energy metabolism [36, 37]. Given the heavy reliance of neurons on mitochondrial function, such dysfunction has particularly profound implications for neuronal health [38].

Consistent with this hypothesis, our study found that MS patient derived fibroblasts displayed mitochondrial dysfunction associated with disorganization of the mitochondrial network.

### The role of oxidative stress in the pathogenesis of MS

Inflammation is a key pathological feature in MS, evident from the earliest stages of disease development [39]. Immune cells penetrate the blood-brain barrier and initiate neuroinflammation, which becomes chronic over time. Oxidative stress, which results from the production of ROS in the inflammatory foci, plays a critical role in exacerbating inflammation in MS. Macrophages and microglia, during the phagocytosis of myelin in white matter, are known to produce ROS, further contributing to oxidative stress [40].

Markers of oxidative stress, such as lipid peroxidation products and protein carbonyls, as well as oxidative DNA damage markers such as 8-hydroxydeoxyguanosine, have been identified in demyelinating lesions of MS, underscoring the widespread nature of oxidative stress in the disease [41].

Mitochondrial dysfunction in MS lesions has been hypothesized to result from oxidative damage to mitochondrial DNA and impaired activity of mitochondrial enzymes, which disrupts oxidative phosphorylation (OXPHOS) and increases ROS production [36]. Because of this, oxidative stress sets in within neurons and glial cells, causing harm to intracellular proteins, lipids, and DNA as well as the emergence of secondary metabolites that may serve as extra autoantigens. Furthermore, ROS directly harm the myelin sheath, promoting the release of new autoantigenic particles that heighten autoimmune inflammation and eventually harm neuronal structures [41, 42]. Thus, it has been proposed that the axonal degeneration associated with myelin loss in demyelinating diseases such as MS is related to oxidative stress caused by impaired OXPHOS [43].

Furthermore, it has been reported that when treated with hydrogen peroxide, MS skin fibroblasts had reduced cell survival rates compared to both Amyotrophic Lateral Sclerosis and control cells suggesting that processes controlling oxidative stress in MS skin fibroblasts were altered [31]. Furthermore, mitochondrial and glycolytic metabolic functions in MS skin fibroblasts were perturbed compared to control cells, which is often associated with increased oxidative stress and altered biological processes [31].

Furthermore, disruption of metabolic pathways results in an imbalance of neurotrophic substances for oligodendrocytes and neurons, resulting in increased axonal demyelination [44].

### Iron/lipofuscin/lipid peroxidation and MS

Mitochondrial dysfunction and increased ROS production also contribute to lipid peroxidation and iron accumulation, leading to cell death by ferroptosis [15]. In fact, the presence of elevated iron levels and abnormalities in iron metabolism have been observed in the brains, spinal cords, and neurons of MS patients, suggesting that ferroptosis plays a significant role in MS pathogenesis [45–47]. Studies show a connection between this type of cell death and MS as well as other disorders of the nervous system [46, 48]. Lipid peroxidation, a hallmark of MS, is closely linked to iron accumulation [49], which exacerbates oxidative damage to cellular structures, particularly mitochondria, and disrupts iron homeostasis, culminating in cell death [46, 50]. Furthermore, it has been demonstrated that several ferroptosis-related genes, such as *Ataxia Telangiectasia Mutated* (*ATM*), *Glycogen Synthase Kinase 3 Beta* (*GSK3B)*, *3-Hydroxy-3-Methylglutaryl-Coenzyme A Reductase* (*HMGCR*), *Kruppel-Like Factor 2* (*KLF2*), *Mitogen-Activated Protein Kinase 1* (*MAPK1*), *Nuclear Factor Erythroid 2 Like 1* (*NFE2L1*), *Neuroblastoma RAS Vital Oncogene Homolog* (*NRAS*), *Poly(RC) Binding Protein 1* (*PCBP1*), *Phosphatidylinositol-4,5-Biphosphate 3-Kinase Catalytic Subunit Alpha* (*PIK3CA*), *Ribosomal Protein L8* (*RPL8*), and *Voltage-dependent anion channel 3* (*VDAC3*), have been associated with MS and may have a potential diagnostic value [23].

In fact, given the high content of iron in neurons, dysregulated iron homeostasis is known to contribute to neurodegenerative diseases such as MS [51]. Iron transport and storage in the body is a complex process that involves several stages [52]. Iron absorption occurs in the duodenum and, once in the blood, iron is bound to the transport protein transferrin (Tf). When the iron-Tf complex arrives to a cell membrane, it is recognized by the TfR and internalized into the cell. Subsequently, iron is reduced to the ferrous ion by the endosomal reductase STEAP3 and transported into the cytoplasm by DMT1. Once in the cytoplasm, iron forms part of the LIP in its ferrous form, from where it can be exported through ferroportin, stored in ferritin in the ferric form, or pass into the mitochondria via mitoferrin1 and 2 (Mfrn1, Mfrn2).

Once inside the mitochondria, iron can accumulate in mtFTL or be used for the formation of iron-sulfur centers. Several proteins participate in this process, including ISCU, LYRM4, FXN, and NFS1 [53]. In our study, the analysis of proteins related to iron metabolism revealed marked alterations. Specifically, the expression levels of IRP-1, TfR, DMT1, FTL, ARA70, Mfrn2, and FXN were downregulated while the expression levels of mtFTL, NFS1, ISCU, and LYRM4 were upregulated. These changes suggest dysregulated iron handling within the cell and mitochondria. In fact, our findings indicate that the altered iron metabolism observed in MS patient-derived cells is closely linked to mitochondrial dysfunction. Mitochondria are not only responsible for energy production but also play a key role in regulating cellular iron homeostasis, particularly through the synthesis of iron-sulfur clusters. Our results show that the dysregulated expression of proteins such as mtFTL, NFS1, ISCU, FXN, and LYRM4, critical for iron-sulfur cluster synthesis, may hinder mitochondrial iron processing and impair cellular energy production, given the essential role these cofactors play in the mitochondrial electron transport chain. Furthermore, LIP levels were decreased indicating that, while iron accumulates in the mitochondria, the available free iron is paradoxically low, potentially impairing mitochondrial function. Additionally, the mitochondrial dysfunction caused by disrupted iron handling could lead to increased ROS production, exacerbating oxidative damage to mitochondrial membranes and triggering lipid peroxidation. These alterations in mitochondrial iron metabolism may contribute to the pathophysiology of MS by amplifying cellular stress and promoting neurodegeneration.

Iron is highly reactive and can catalyze the formation of phospholipid peroxyl radicals, leading to lipid peroxidation and, ultimately, ferroptosis, a programmed cell death characterized by iron-dependent oxidative damage and subsequent plasma membrane rupture due to a redox imbalance between oxidants and antioxidants [54]. In fact, we demonstrated that if we eliminate iron accumulation by deferiprone supplementation, we reduce lipofuscin-like aggregates and lipid peroxidation in MS fibroblasts.

On the other hand, it has been shown that GSH levels are reduced in the cerebrospinal fluid of MS patients and that GPX4 activity is affected during MS pathology [3]. All this causes a greater susceptibility to death by ferroptosis. There are classical ferroptosis activators such as erastin or RAS-selective lethal 3 (RSL3) [54]. Erastin is a small molecule capable of initiating ferroptosis, inhibiting cystine import via the cystine/glutamate antiporter system Xc- required for exchange with intracellular glutamate [55].

Our results showed that MS cells have a great susceptibility to erastin treatment suggesting that both iron accumulation and lipid peroxidation are underlying pathological mechanisms in MS. In addition, iron overload stimulates lipid peroxidation which causes more iron accumulation in a vicious cycle [15].

### Autophagy and MS

Our results also showed impaired lysosomal acidification, leading to autophagosomes accumulation and increased expression levels of LAMP1, a membrane lysosomal marker, as well as increased autophagy-related proteins expression levels. These results suggest that autophagosomes and lysosomes are accumulated because lysosomal acidification deficiency disrupts autophagy process. Autophagy has the property of a double-edged sword in MS in that it may have both beneficial and detrimental effects on MS neuropathology [56]. Autophagy prevents the progression of MS by reducing oxidative stress and inflammatory disorders. In contrast, excessive autophagy activation is associated with the progression of MS neuropathology. In such cases, the use of autophagy inhibitors may alleviate MS pathogenesis [56].

### Inflammasome and MS

Mitochondrial dysfunction is also linked to inflammasome activation, particularly the NLRP3 inflammasome, which plays a crucial role in the inflammatory response observed in MS [34].

The NLRP3 inflammasome is a multiprotein complex of the innate immune system that contributes to the pathogenesis of MS by regulating the production of pro-inflammatory cytokines (IL1B and IL-18) and the induction of pyroptotic cell death. Mitochondrial dysfunction is one of the main potential factors that can trigger NLRP3 inflammasome activation and lead to inflammation and axonal damage in MS. This highlights the importance of understanding how mitochondria modulate NLRP3 inflammasome activity and contribute to the inflammatory and neurodegenerative features of MS [57]. Several lines of evidence suggest an association between inflammasome activation and MS pathogenesis [58–60]. Additionally, genetic polymorphisms in NLRP3-related genes have been associated with MS susceptibility and severity, further highlighting the role of the inflammasome in MS [61–63]. Moreover, studies have reported increased expression levels of NLRP3 and IL1B genes in MS plaques and elevated levels of caspase-1 and IL-18 in the sera of MS patients [64]. As the downstream effectors of the NLRP3 inflammasome, IL1B and IL-18 can be used as potential biomarkers for MS.

Our results showed that NLRP3 inflammasome is activated in MS fibroblasts associated with caspase-1 activation and increased production of IL1B.

In summary, we observed pathophysiological alterations in fibroblasts derived from MS cells, including cellular senescence, mitochondrial dysfunction, iron/lipofuscin accumulation, and inflammasome activation. These alterations mirror those found in patients with the disease, suggesting that fibroblasts from MS patients could serve as a valuable cellular model for studying the disease. Moreover, these alterations are common features of aging. However, the accelerated aging observed in MS patients reflects a unique interplay of disease-specific mechanisms that exacerbate these processes beyond what is typically seen in normal aging. Chronic systemic inflammation, immunosenescence, and the release of pro-inflammatory cytokines amplify neuroinflammatory and neurodegenerative pathways, contributing to the premature manifestation of age-related biological markers such as DNA methylation changes [65]. While some molecular changes, such as telomere shortening and mitochondrial dysfunction, may result from MS-related stress, others, such as aged microglial dysfunction and pro-inflammatory feedback loops, may predispose patients to disease progression, creating a bidirectional relationship between MS pathology and accelerated aging [66]. This interplay highlights how MS-specific factors intensify aging-related mechanisms.

## CONCLUSIONS

In this work, we provide evidence that fibroblasts derived from MS patients manifest pathophysiological alterations suggesting that altered underlying molecular mechanisms may be the origin of neuroinflammation in MS. Mitochondrial dysfunction, iron/lipofuscin accumulation, lipid peroxidation, inflammasome activation and increased expression of proinflammatory cytokines in cells from MS patients may explain the subjacent cellular damage and be the origin of the subsequent chronic pathological overactivation of immune system cells. MS cellular models can be highly useful for the identification of dysregulated cellular pathways, which may elucidate the etiopathogenesis of MS.

## MATERIALS AND METHODS

### Reagents

Anti-Mitochondrially Encoded Cytochrome C Oxidase Subunit II (mtCO2) (ab170681), anti-Cytochrome C Oxidase subunit IV (COX IV) (ab14744), anti-mitochondrial ferritin (MtFTL) (ab124889), anti-LYR motif-containing protein 4 (LYRM4) (ab253001), anti-Divalent Metal Transporter 1 (DMT1) (ab55735), anti-ATP synthase F1 subunit alpha (ATP5F1A) (ab14748), anti-Voltage-dependent anion channel (VDAC) (ab14734), anti-frataxin (FXN) (ab219414), anti-caspase 1 (ab179515), anti-Androgen Receptor Activator 70 (ARA70) (ab86707), anti-NFS1 cysteine desulfurase (NFS1) (ab58623), anti-manganese superoxide dismutase (MnSOD) (ab68155), Goat Anti-Rabbit IgG H&L (HRP) (ab6721), Rabbit Anti-Mouse IgG H&L (HRP) (ab6728), and Rabbit Anti-Goat IgG H&L (ab6741) were purchased from Abcam (Cambridge, UK).

Anti-NADH:ubiquinone oxidoreductase core subunit S1 (NDUFS1) (PA5-22309), anti-NRL family pyrin domain containing 3 (NLRP3) (PA5-20838), anti-interleukin 1-beta (IL1B) (PA5-68046), anti-glutathione peroxidase 4 (GPX4) (MA5-32827), Mitotracker™ Red CMXROS (M46752), anti-Iron Sulfur Cluster Assembly Enzyme (ISCU) (MA5-26595), anti-NADH:ubiquinone oxidoreductase subunit A9 (NDUFA9) (459100), anti-Transferrin Receptor (TfR) (13-6800), anti-mitoferrin 2 (Mfrn2) (12703), DAPI (D1306), Bovine Serum Albumin (BSA) (BP7902), Hoescht (10150888), Lysotracker™ Green DND-26 (L7526), and propidium iodide (PI) (11539226) were purchased from Invitrogen™/Molecular probes (Eugene, OR, USA).

Anti-Iron-Responsive Element-Binding Protein 1 (IRP-1) (sc-166022), anti-ferritin light chain (FTL) (sc-74513), anti-Lysosomal Associated Membrane Protein 1 (LAMP1) (sc-20011), anti-Autophagy Receptor p62 (p62) (sc-48402), anti-superoxide dismutase 1 (SOD1) (sc-101523), oligomycin (sc-203342), rotenone (sc-203242), antimycin A (sc-202467A), deferiprone (sc-211220), carbonyl cyanide ptrifluoromethoxy-phenylhydrazone (FCCP) (sc-203578), and BODIPY® 581/591 C11 (D3861) were purchased from Santa Cruz Biotechnology (Dallas, TX, USA).

Anti-actin (MBS448085) was purchased from MyBioSource (San Diego, CA, USA). Phosphate-buffered saline (PBS) (102309) was purchased from iNtRON Biotechnology (Seongnam, Republic of Korea). Anti-Microtubule-Associated Protein 1A/1B Light Chain 3B (LC3B) (2775S) and anti-Autophagy-Related 12/Autophagy-Related 5 (Atg12/5) (2010S) were purchased from Cell Signaling (Danvers, MA, USA). Paraformaldehyde (PFA) (158127), dimethyl sulfoxide (DMSO) (17093), Prussian Blue (03899), Luperox® DI (168521), vitamin E (T3251), and Sudan Black B (199664) were purchased from Sigma-Aldrich (Saint Louis, MO, USA).

### Cell culture

We used primary skin fibroblasts from nine patients with MS, who presented clinical and radiological evidence compatible with the disease, and from six unaffected individuals:

Samples from patients and controls were obtained according to the Helsinki Declarations of 1964, as revised in 2001.

Patients and controls’ fibroblasts were cultured at 37° C and 5% CO_2_ in DMEM glucose (Dulbecco’s Modified Eagle Medium) supplemented with 10% Fetal Bovine Serum (FBS) and 1% Penicillin/streptomycin (Thermo Fisher Scientific, Waltham, MA, USA).

**Table:** 

**PATIENT**	**DIAGNOSIS**	**SEX**	**AGE**
**C1**	Unaffected	F	40
**C2**	Unaffected	F	39
**C3**	Unaffected	F	42
**C4**	Unaffected	F	41
**C5**	Unaffected	F	50
**C6**	Unaffected	F	48
**P1**	RRMS	F	47
**P2**	RRMS	F	33
**P3**	RRMS	F	46
**P4**	SPMS	F	42
**P5**	RRMS	F	45
**P6**	SPMS	F	51
**P7**	RRMS	F	40
**P8**	SPMS	F	40
**P9**	RRMS	F	49

All experiments were conducted using fibroblasts at a passage number of less than 10.

### Cellular morphology analysis

For the analysis of cellular morphology, light microscopy was used. The cell area was measured using Fiji-ImageJ software version 1.53.2.

### Determination of iron and lipofuscin accumulation

Iron accumulation was determined by Perl's Prussian Blue staining [67]. Images were taken by light microscopy using an Axio Vert A1 microscope (Zeiss, Oberkochen, Germany) and analyzed by Fiji-ImageJ software version 1.53.2.

In addition, iron content was quantified in cell culture extracts, obtained from acid digestion using nitric acid, by inductively coupled mass spectrometry (ICP-MS), which was performed with an Agilent 7800 spectrometer (Agilent Technologies, Santa Clara, CA, USA).

Lipofuscin accumulation was assessed by Sudan Black B staining [68, 69]. Images were acquired by light microscopy using an Axio Vert A1 microscope (Zeiss, Oberkochen, Germany) and analyzed by Fiji-ImageJ software version 1.53.2. Autofluorescence was evaluated by fluorescence microscopy with a Nikon A1R confocal microscope (Nikon, Shinagawa, Tokyo, Japan). Confocal laser scanning microscopy was used to obtain the emission spectra of lipofuscin granules.

### Determination of labile iron pool (LIP)

To determine the Labile Iron Pool (LIP), cells were seeded in 12-well plates for 24 hours in DMEM glucose. Cells were then incubated in the medium supplemented with 0.25 μM Calcein-AM at 37° C for 15 minutes. After that, cells were washed twice with Hank′s Balanced Salt Solution (HBSS) and then incubated in HBSS supplemented with 10 mM glucose for 10 minutes. At that time, basal fluorescence was measured using a Polar Star Omega Microplate Reader (BMG Labtech, Offenburg, Germany). Subsequently, cells were treated with the iron chelator deferiprone (100 μM) for 15 minutes. Fluorescence was monitored during this incubation, and when a plateau was reached, this value was recorded as the LIP. Results were normalized to protein content.

### Immunoblotting

Western Blotting assays were performed using standard methods. Proteins were transferred to nitrocellulose membranes, which were then incubated with primary antibodies at a proper dilution range (1:500-1:2000) overnight at 4° C. Subsequently, membranes were incubated with the corresponding secondary antibodies coupled to horseradish peroxidase (HRP) at a 1:2500 dilution for 1 hour at room temperature. ChemiDoc™ MP Imaging System (BioRad, Hercules, CA, USA) was used to reveal protein signals. Results were normalized to housekeeping protein actin and analyzed by ImageLab™ software version 5.0 (Biorad, Hercules, CA, USA).

### Analysis of mitochondrial network

The mitochondrial network was analyzed using Mitotracker™ Red CMXROS (100 nM, 45 min, 37° C). After that, cells were incubated with 1 μg/mL DAPI for 10 minutes. Images were acquired using a DeltaVision System (Applied Precision, Issaquah, WA, USA) and analyzed by Fiji-ImageJ software version 1.53.2.

### Lysosome acidification

The analysis of lysosomal compartment was performed using Lysotracker™ Green DND-26 staining. Lysotracker is an acidotropic dye that stains cellular acidic compartments, including lysosomes and autolysosomes. Cells were incubated with 75 nM LysoTracker™ Green DND-26 (L7526, Thermo Fisher Scientific, Waltham, MA, USA) for 1 hour. Images were acquired using a DeltaVision System (Applied Precision, Issaquah, WA, USA) and analyzed by Fiji-ImageJ software version 1.53.2.

### Mitochondrial bioenergetics

Mitochondrial respiratory function of control and patients’ fibroblasts was assessed using the Mitostress test assay using a XFe24 extracellular flux analyzer (Seahorse Bioscience). Basal respiration, maximal respiration, spare respiratory capacity, and ATP production were quantified by measuring the oxygen consumption rate (OCR; pmol O_2_/min), normalized to cell number, after the sequential injection of oligomycin, FCCP and rotenone/antimycin A. A minimum of five wells per experimental condition were utilized.

### Statistical analysis

Statistical analysis was performed as previously reported by our study team [70]. When the number of events was low (n<30), we employed non-parametric statistics without any distributional assumptions. In these instances, the Kruskal-Wallis test was used to compare different groups. For a higher number of events (n>30), parametric testing was applied. In these cases, a one-way ANOVA was used for group comparisons. The GraphPad Prism (version Prism 10.0.2) (GraphPad Software, San Diego, CA, USA) software was used to perform statistical analysis. Data are presented as the mean ± SD of at least three independent experiments. p-values below 0.05 were considered significant.
